# The Effect of Vaccination with *Neospora caninum* Live-Frozen Tachyzoites on Abortion Rates of Naturally Infected Pregnant Cows

**DOI:** 10.3390/vaccines9040401

**Published:** 2021-04-19

**Authors:** Monica L. Mazuz, Benjamin Leibovitz, Igor Savitsky, Elena Blinder, Daniel Yasur-Landau, Yaniv Lavon, Binyamin Sharir, Sharon Tirosh-Levy

**Affiliations:** 1 Division of Parasitology, Kimron Veterinary Institute, Beit Dagan 50200, Israel; borisl@moag.gov.il (B.L.); igors@moag.gov.il (I.S.); elenab@moag.gov.il (E.B.); DanielY@moag.gov.il (D.Y.-L.); sharontirosh@gmail.com (S.T.-L.); 2 Israel Cattle Breeders Association, Caesarea 38900, Israel; yaniv@icba.co.il; 3 Hachaklait Veterinary Services, Caesarea 3039, Israel; sharir@hak.org.il; 4 Koret School of Veterinary Medicine, The Robert H. Smith Faculty of Agriculture, Food and Environment, The Hebrew University of Jerusalem, Rehovot 7610001, Israel

**Keywords:** *N. caninum*, neosporosis, abortion, cattle, vaccination, control

## Abstract

Neosporosis is a major cause of abortions in cattle worldwide. Recently a live attenuated vaccine showing promising results in preventing abortions, when administered at mid-pregnancy to seropositive cows, was developed. In this study, vaccination with 2 × 10^8^ live frozen *N. caninum* tachyzoites (NcIs491) was used to immunize naturally infected seropositive pregnant dairy dams. The study was performed under field conditions in four herds, and a follow-up of three subsequent pregnancies was analyzed. A total of 1136 cows were serologically examined. Total seroprevalence was 41.4%, with 25.1% of the cows having titers of 1:800 or higher. Abortion rates were significantly higher in cows with high antibody titers (≥1:800) for two consecutive pregnancies. Vaccination was administered to 114 out of 285 cows with antibody titers higher than 1:800. Immunization resulted in lower abortion rates at three of the farms. Vaccine efficacy ranged from −19.8% to 75% at different farms, with overall efficacy of 28.4% in all four farms and overall efficacy of 58.2% in the three farms with positive results. Our results showed different vaccine efficacy in studied farms, suggesting that frozen live vaccination may generally be an effective method to control neosporosis in cattle.

## 1. Introduction

*Neospora canimum* is an intracellular apicomplexan parasite affecting various animal species, and a leading cause of abortion in cattle worldwide [1,2,3]. The parasite has been reported from most parts of the world, with varying prevalence between areas and farms, which may reach up to 97% (reviewed in [1]). Most infected cattle remain asymptomatic and, apart from abortion, no clinical signs have been reported [1]. It has been demonstrated that *N. caninum-*carrier dams have an increased risk of repetitive abortions in consecutive pregnancies [4], which has considerable economic consequences [2].

Infection may be the result of ingestion of oocysts secreted by the definitive canid host [1,3], however, transplacental transmission is very efficient for this parasite [3], and considered as its major route of transmission in cattle [4,5,6]. Due to the combination of efficient vertical transmission and high prevalence in some cattle herds, prevention and control of neosporosis is challenging. 

In *N. caninum* infection, immune protection is mainly cell-mediated rather than humoral [7]. Cell-mediated immune mechanisms have a major function in controlling neosporosis in cattle [8], while humoral response is not protective [7]. The presence of specific antibodies is indicative of parasite exposure and seropositivity is associated with higher risk of abortions [4]. The antibody titer is directly associated with the chance of vertical transmission, with seropositivity remaining for years. 

Currently, there is no effective chemotherapy or commercially available vaccine. A commercial inactivated vaccine (Neoguard^®^, Intervet International B.V., Boxmeer, The Netherlands) was withdrawn from the market, as only moderate protection against abortions was observed in field trials [9,10]. Previous studies demonstrated that vaccination with specific selected antigens, tachyzoite lysate or live tachyzoites induced protection against experimental challenges in mice and cattle [11,12,13,14,15,16]. However, live vaccine, mainly with isolates of low pathogenicity, is considered as the most promising and efficient prophylactic measure [14,17,18].

The Israeli *N. caninum* isolate (NcIs491), obtained from brain tissues of an aborted fetus, was cultured and found to have low pathogenicity in laboratory animals [19], making it a suitable vaccine candidate. Recently, a fresh live vaccine with the Israeli strain NcIs491 has been developed, with an efficacy of 39% in preventing abortions in seropositive cows under field conditions [20]. Despite its effectiveness, the use of this fresh-live vaccine in-field has considerable limitations. Fresh parasites are only viable for a few days in the refrigerator; consequently, the logistics of its production, dispatch and use by attending veterinarians is problematic on a large scale. Particularly as this protocol of vaccination should be performed in a very determined period (between 120 to 140 days of pregnancy), and it is not suitable for the vaccination of an entire herd at once. Therefore, a frozen live vaccine is desired for use in the field, as it survives longer, permitting large-scale production, conservation, and availability. 

In this study, we tested the efficacy of a frozen live vaccine of the same strain. The study was conducted in field conditions in four different *Neospora*-endemic dairy cattle herds. Additionally, the outcome of three consecutive pregnancies after a single vaccination was recorded in order to evaluate the long-term effect of vaccination. 

## 2. Materials and Methods 

### 2.1. Study Design

The farms selected to participate in this trial were randomly chosen from farms with the following requirements: (1) A history of neosporosis, with recorded prevalence higher than 30%; (2) the use of computerized monitoring of the animals; and (3) willingness of both farmers and attending veterinarians to participate. Four dairy cattle farms endemic with neosporosis were included in the study. The study population comprised mostly of first-calf heifers that were sampled during the period 2015–2017. Cows were screened for serologic exposure to *Neospora* spp. Using an indirect fluorescent antibody test (IFAT) in days 110–120 of pregnancy. Only seropositive animals with antibody titers higher than 1:800 were included in the study. 

The study population of seropositive heifers in each farm was randomly divided into two groups. Group A was vaccinated with *N. caninum* live frozen tachyzoites on days 120–140 of pregnancy, while group B served as unvaccinated controls and no treatment was administered. The outcome of all pregnancies was recorded for both groups, and, when possible, the outcome of sequential pregnancies was also recorded. The analyses of further pregnancies were performed without retesting the sero-status of the dams, and with no additional vaccination. 

The study was conducted upon owner’s consent and approved by the Animal Experiments Welfare Committee of the Kimron Veterinary Institute (b-8153-3-15).

### 2.2. Sample Collection and Serological Screening

Pregnancy tests were performed on days 110 to 120 after insemination by the fetal membrane-slip method, and blood was collected from the tail blood vessels of all pregnant cows. Serum was obtained after centrifugation at 4000× *g* for 4 min and tested for the presence of anti-*Neospora* spp. antibodies by an immunofluorescence antibody test (IFAT), as previously described [21].

### 2.3. Vaccination Procedure

All heifers in group A were vaccinated with *Neospora caninum* live tachyzoites administered subcutaneously on days 120–140 post-insemination. Parasite culture and vaccine preparation was performed as previously described [20]. Each dose of inoculum contained 2 × 10^8^ parasites and was kept frozen in liquid nitrogen until use.

### 2.4. Statistical Analysis

The association between abortion and the presence and titer of anti-*Neospora* antibodies was evaluated based on the population of nonvaccinated cows in the first fertility cycle, using Fisher’s exact test and odds ratio (OR). The correlation between antibody titer and abortion rate was estimated by spearmen’s rho (ρ). The distribution of repeated abortions was compared between groups using independent samples Kruskal–Wallis test.

The effect of vaccination was evaluated in the population of cows with anti-*Neospora* antibody titers higher than 1:800, using Fisher’s exact test and odds ratio (OR). To control the farm effect, the association of vaccination against abortion, was also analyzed using generalized estimating equation (GEE) using logit link function, with the cow defined as the subject and the farm defined as a random variable. Vaccine efficacy (VE) was calculated using the formula: VE = (ARU–ARV)/ARU × 100 (ARU = abortion rate in unvaccinated cows, ARV = abortion rate in vaccinated cows). 

Statistical significance was set at *p* < 0.05. The analysis was performed using SPSS 22.0^®^ (IBM Corp, Armonk, NY, USA) and Win Pepi 11.43^®^ statistical software(Abramson, J.H. WINPEPI updated: computer programs for epidemiologists, and their teaching potential. Epidemiologic Perspectives & Innovations, 2011, 8:1).

## 3. Results

### 3.1. Study Population and Initial Screening

A total of 1136 cows from four dairy farms were screened for serologic exposure to *Neospora* spp. Between 120 and 512 cows were tested at each farm. Most cows (*n* = 950, 83.6%) were first-calf heifers, some had two previous calvings (*n* = 167, 14.7%), while the rest (*n* = 19, 1.7%) had calved three-to-five times. 

**Figure 1:**
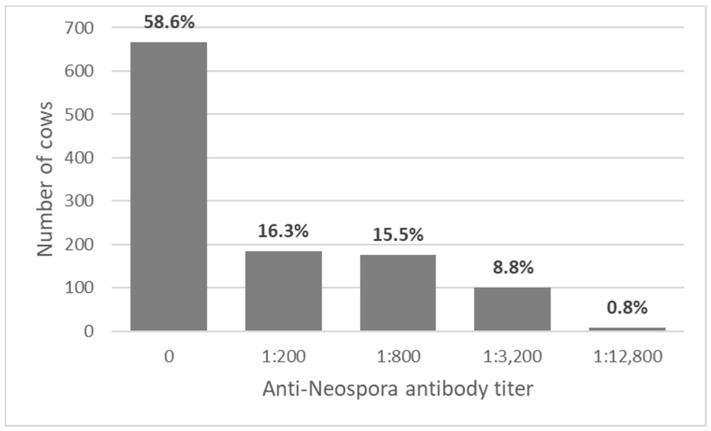
The distribution of anti-*Neospora* antibody titers of 1136 cows from four dairy farms, as tested by immunofluorescence antibody test (IFAT).

*Neospora* spp. seroprevalence in the study population was 41.4% (*n* = 470). The seroprevalence varied between farms and ranged between 29.9% and 57.5% (*p* < 0.001). Antibody titers were 1:800 or higher in 25.1% of the cows (*n* = 285) (Figure 1), and anti-*Neospora* vaccination was administered to 114 of them.

### 3.2. Neospora as a Cause of Abortion

**Figure 2:**
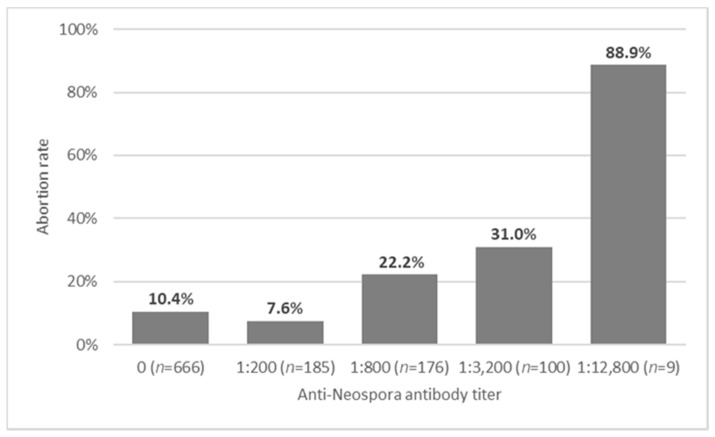
Abortion rates in 1020 unvaccinated cows from four dairy farms, in relation to their anti-*Neospora* antibody titers, as tested by immunofluorescence antibody test (IFAT).

The overall abortion rate in the study population was 14.2% (161 of 1136 pregnancies), and was 13.3% in unvaccinated cows (136 of 1022 pregnancies, Table 1). When excluding the vaccinated cows, abortion rate was significantly higher in *Neospora* seropositive cows, with an antibodies titer of 1:200 or higher (67/356, 18.8%) than in seronegative cows (69/666, 10.4%) (odds ratio (OR) = 2.01, 95% confidence interval (CI): 1.37–2.93, *p* < 0.001). However, cows with low anti-*Neospora* antibody titer (1:200) did not differ from seronegative cows in their abortion rates (*p* = 0.326), and the rate of abortions increased with anti-*Neospora* antibody titers (ρ = 0.167, *p* < 0.001, Figure 2), as well as with the number of past pregnancies (ρ = 0.107, *p* = 0.001, data not shown).

The rate of abortions increased in subsequent fertility cycles in the study population (excluding vaccinated animals), regardless of their *Neospora* status, from 13.3% to 20.6% (ρ = 0.075, *p* < 0.001, *n* = 2286, Table 1). The abortion rate was also associated with the number of past calvings (ρ = 0.087, *p* < 0.001, *n* = 2286, data not shown). 

Abortion rates in unvaccinated cows with high (≥1:800) anti-*Neospora* antibody titers were significantly higher than in seronegative cows (OR = 3.82 95% CI: 2.48–5.85, *p* < 0.001, Table 1), and in seronegative or suspected (≤1:200) cows in the first two reproduction cycles (30.6% versus 9.8%, *p* < 0.001 and 23.4% versus 15.2%, *p* = 0.037 respectively), but not in the third (29% versus 19.4%, *p* = 0.093). Odds ratios for abortion in cows with high (≥1:800) antibody titers in relation to cows with antibody titers equal or lower than 1:200 were 4.08 (95% CI: 2.68–6.15), 1.71 (95% CI: 1.0–2.83), and 1.7 (95% CI: 0.88–3.18) in the first, second, and third pregnancies. In the second observed pregnancy, the rate of abortions in suspected cows (titer of 1:200) was significantly higher than in seronegative cows in farm 2 (29.4% versus 5.3%, *p* = 0.024), and in the entire study population (13.5% versus 21.2%, *p* = 0.026).

A total of 555 cows were available for follow-up for three reproduction cycles. In these cows, the distribution of the cumulative number of abortions per dam was significantly different in positive dams with high antibody titer (≥1:800) compared to seronegative dams (Table 2). The distribution of abortion in cows with titer of 1:200 or in seropositive vaccinated cows did not differ from seronegative dams. The overall abortions from all pregnancies of high seropositive cows were higher than in seronegative cows (*p* = 0.058, Table 2), although the difference was not statistically significant. Low rates of repeated abortions were observed in all groups, with a total of 3.8% of unvaccinated cows.

### 3.3. Anti-Neospora Vaccination and Abortion Rates

In the vaccinated pregnancy, the abortion rate in vaccinated and unvaccinated seropositive cows (with antibody titers ≥ 1:800) was 21.9% and 30.6%, respectively (OR = 0.64, 95% CI: 0.35–1.13, *p* = 0.136, Table 3). Reduction in abortion rates in vaccinated cows was observed in three out of four farms, which was statistically significant in farm 3 (Table 3). Vaccine efficacy in preventing abortions was 28.43%, and varied between farms (−19.86 to 75%, Table 3). In farm 4, a negative, non-significant impact was observed, and the rate of abortion was higher in vaccinated cows (35.6%) compared to seropositive non vaccinated ones (29.7%, *p* = 0.539). When farm 4 was removed from the analysis to better evaluate the positive effect of the vaccine, significantly lower abortion rates were noted in vaccinated cows compared to unvaccinated ones (13.04 versus 31.19, *p* = 0.007), and vaccine efficacy was calculated as 58.2% (*p* = 0.007). Farms did not differ significantly in total abortion rates (*p* = 0.839) nor in abortion rates in cows with high anti-*Neospora* antibody titers (>1:800, 0.334). A significant difference in abortion rates between farms was only observed in the vaccinated group (*p* = 0.043). When the farm was controlled as a confounding effect by using a multivariable model, the effect of the vaccine was more pronounced, although still not significant when analyzing all four farms together (*p* = 0.094, OR = 0.62 95%CI: 0.36–1.08), and highly significant for farms 1–3 (*p* = 0.006, OR = 0.32, 95% CI: 0.14–0.72).

The follow-up of two consecutive pregnancies after vaccination resulted in similar vaccine efficacy in sequential pregnancies (Table 3). A significantly lower abortion rate in farm 3 was also observed in the second, nonvaccinated, pregnancy (Table 3). Total abortion rates in two further pregnancies after vaccination showed a reduction in abortions in vaccinated animals (*p* < 0.001, Table 3). Overall, abortions in all three reproduction cycles were significantly lower in vaccinated than in unvaccinated cows (21% versus 28%, respectively, *p* = 0.048), and vaccine efficacy was 25.1% for all three cycles after single vaccination. When farm 4 was removed from the analysis, overall abortion rates in farms 1–3 in all three cycles were 13.8% and 27.8% in vaccinated and unvaccinated cows, respectively (*p* = 0.001), and vaccine efficacy was calculated as 50.2%. 

The number of cows available for follow-up reduced from 1136 in the initial screening to 555 by the third fertility cycle (a reduction of 51.1%). This reduction was similar in the seronegative, suspected, and vaccinated groups (a reduction of 48%, 52.4% and 47.4%, respectively), but was significantly higher in the nonvaccinated group (64.2%, *p* < 0.001).

## 4. Discussion

Vaccination of pregnant heifers with *Neospora caninum* live tachyzoites had been previously tested, showing promising results [20]. The current study was set to test vaccine efficacy in four different farms and to evaluate the use of frozen live vaccine, instead of fresh live vaccine. The ability to use frozen vaccine is important for making it feasible for use in the field by clinicians in the future. The results of this study demonstrate that the use of frozen inoculum consisting of 2 × 10^8^ live tachyzoites, thawed in the field and injected immediately, has similar efficacy to 10^8^ parasites in fresh, nonfrozen, vaccine [20]. Vaccine administration in the field was similar to other live attenuated cattle vaccinations currently in use (*Babesia bovis*, *Babesia bigemina*, *Theileria annullata*), and could be easily implemented by attending veterinarians [22,23,24].

The overall prevalence of anti-*Neospora* antibodies in the study population was 41.4% (95% CI: 38.9–43.8%) and varied between farms (29.9–57.5%), similar to previous reports from the area (51.4% and 35.5%) [4,25]. In the tested pregnancy, the abortion rate of *Neospora*-seropositive dams was higher than in seronegative cows, and correlated with antibody titer, as previously reported [4,26]. The rate of abortions did not differ between cows with antibody titers of 1:200 and seronegative cows in the tested pregnancy. However, in the following, pregnancy abortion rates were significantly higher in the “suspected” (titer of 1:200) group than in the negative group (OR = 1.73, 95% CI: 1.04 to 2.83, *p* = 0.026). As previously noted, in endemic herds, titers of 1:200 should be considered borderline (suspected) and unstable [4]. Increased post-partum antibody titers were observed in over one-third of cows tested as “suspected” in mid-pregnancy [4]. Since in this study, antibody titers have only been tested during the first inspected pregnancy; the relative increase in abortions in the second pregnancy of the “suspected” group may reflect an increase in antibody titer or de-novo infection in some of the cows. The significance of seropositivity with titers of 1:200 in term of control approaches should be evaluated separately. Hence, in this vaccination trial, as well as in the previous [20], only cows with antibody titers of 1:800 and higher were eligible for vaccination.

Previous studies had shown that, in cows vaccinated with live tachyzoites before pregnancy, a challenge at mid-gestation induced strong and long-lasting specific interferon (IFN-gamma) response, sufficient to protect the fetus and prevent abortion [11,14,27]. However, since cellular immune assays could not be performed under field conditions, we presume that vaccination of infected dams with live tachyzoites during pregnancy acts as a challenge, inducing a specific immune response capable of detaining the multiplication of parasites and, consequently, protecting against abortion [20]. 

Vaccine efficacy in preventing abortions was estimated as 28% when analyzing all four farms together. Although the overall efficacy was lower than in the previous vaccine trial using fresh tachyzoites (39%, [20]), a significant difference between vaccine efficacy was observed between farms, ranging from −19.86% to 75%. When the farms were analyzed separately, a significant reduction of the abortion rate was observed in one farm (farm 3), and the trend was similar on two additional farms. Thus, analysis of vaccine efficacy in these 3 farms was 58.2% (*p* = 0.007). In one farm (farm 4), the abortion rate was higher in vaccinated dams compared to unvaccinated seropositive cows. The main factor that may have contributed to the differences in vaccine efficacy between farms is not fully clarified. Since this vaccine trial was performed under field conditions, and the selection of farms was performed randomly after a call to participate in this experiment, several factors could not have been homogenized between the four participating farms. In addition, as abortions are normally multifactorial and can be influenced by several factors, including stress. We suggest that animals’ stress, incurred by management and husbandry practices, could have had an impact on overall abortion rates [28], and, specifically, on *Neospora* related abortions [29] in some of the farms. We observed that Farm 3 had very good biosecurity practices and good animal movement and milking schedule management, while farm 4 had the poorest management, being more crowded than the other farms, providing the cows fewer opportunities to ruminate and with longer waiting times between milking. We suppose that the wellbeing of the vaccinated cattle is essential to inducing efficient immune responses after vaccination with live tachyzoites. On the contrary, it appears that, on farm 4, the immunological challenge caused by the vaccine may have added to other immunological stress that we could not address, triggering a negative, non-expected, effect of the vaccine. Thus, we suggest that the combination between vaccination and good biological safety and management practice is crucial for the prevention of *Neospora*-associated abortions, while each of these factors alone is insufficient.

In this study, cows were only tested and vaccinated once, and the efficacy of the vaccine was evaluated on three subsequent pregnancies. In our previous vaccine trial [20], protective efficacy of the vaccine was absent in consecutive pregnancies. Here, although not statistically significant in each separate cycle, the trend in the protective effect of vaccination was similar in two further pregnancies, and the overall abortion rate in all three reproduction cycles was significantly lower in vaccinated cows than in seropositive, nonvaccinated cows, suggesting a long-lasting effect. The increase in abortion rates in consecutive pregnancies may be partially attributed the increase of age, regardless of the *Neospora* status, and partially to the additive increase in the chance of abortion in seropositive cows [4]. A significantly higher chance of abortion in seropositive cows was noted in two of the three pregnancies; however, since the cows were not retested in the following cycles, it is possible that some of the negative cows seroconverted or had changes in their antibody titer by the third reproduction cycle. Another factor that may have influenced the results is the fact that seropositive or unvaccinated cows are removed from the herd more often than seronegative or vaccinated cows. The cause of cows being withdrawn was not noted, but it is normally accepted that cows aborting in the dry period are sent for culling. The possibility that vaccination may have influenced the herd managers’ choice of keeping a cow after abortion cannot be excluded, as they were not blinded to treatment.

This study was performed under field conditions in order to evaluate the efficacy of the vaccine in different management situations. Field studies are of great value for the decision-making process before the introduction of a new approach for use on a large scale in the field. However, it incurred several limitations, including the absence of a systematic selection of farms with similar conditions; the inability to evaluate the stress level during pregnancy on different farms; no option to perform post-treatment evaluation by unbiased observers, or evaluation of immunological response upon vaccination; and no possibility to perform screening to exclude other abortion agents. Nevertheless, the use of several farms with different hygiene and management conditions revealed a divergence in vaccine efficacy on different farms, and highlighted potential drawbacks when the vaccine was applied in realistic situations. Although absolute evaluation of the effect of the vaccine could only be performed under laboratory conditions, the results of this trial suggests that the prevention of *Neospora*-related abortions is complex and should include both vaccination and improvement of biosecurity.

## 5. Conclusions

Vaccination of *Neospora*-seropositive cows with live-frozen inoculum during pregnancy proved beneficial in preventing abortions on several farms, principally when combined with good management practices. This combination might be a useful control measure that could be implemented by farm personnel and attending veterinarians. Our data suggest that the protective effect may last for more than one pregnancy. Vaccine efficacy differed between farms and was not protective in one out of four farms vaccinated. Therefore, the use of a live vaccine to control abortion and reduce economic burdens should be individually analyzed per farm, and not used as a generalized recommendation. 

## Figures and Tables

**Table 1:** Abortion rates of cows from four dairy farms according to their *Neospora* serological status in three consecutive reproduction cycles.

Farm	Group	Cyc1 (N)	AR (%)	OR (95% CI)	Sig	Cyc2 (N)	AR (%)	Sig	Cyc3 (N)	AR (%)	Sig
1	Neg	71	10 (14.1)	ref		48	7 (14.6)		32	4 (12.5)	
	Sus	22	1 (4.5)	0.29 (0.01–2.29)	0.449	17	2 (11.8)	1	9	2 (22.2)	0.597
	Pos	27	5 (18.5)	1.39 (0.33–5.06)	0.549	19	5 (26.3)	0.299	11	4 (36.4)	0.172
2	Neg	51	4 (7.8)	ref		38	2 (5.3)		25	5 (20.0)	
	Sus	24	3 (12.5)	1.68 (0.22–10.83)	0.673	17	5 (29.4)	**0.024**	8	1 (12.5)	1
	Pos	27	9 (33.3)	5.88 (1.39–28.75)	**0.008**	15	3 (20.0)	0.131	5	0	0.556
3	Neg	359	39 (10.9)	ref		287	40 (13.9)		196	37 (18.9)	
	Sus	65	6 (9.2)	0.83 (0.28–2.11)	0.829	58	13 (22.4)	0.112	34	4 (11.8)	0.466
	Pos	55	20 (36.4)	4.69 (2.31–9.28)	**<0.001**	30	6 (20.0)	0.411	16	5 (31.3)	0.323
4	Neg	185	16 (8.6)	ref		139	20 (14.4)		93	23 (24.7)	
	Sus	72	4 (5.6)	0.62 (0.15–2.02)	0.604	54	11 (20.4)	0.382	36	8 (22.2)	0.822
	Pos	64	19 (29.7)	4.46 (1.98–10.03)	**<0.001**	47	12 (25.5)	0.116	30	9 (30.0)	0.634
Total	Neg	666	69 (10.4)	ref		512	69 (13.5)		346	69 (19.9)	
	Sus	183	14 (7.7)	0.72 (0.36–1.33)	0.326	146	31 (21.2)	**0.026**	87	15 (17.2)	0.650
	Pos	173	53 (30.6)	3.82 (2.48–5.85)	**<0.001**	111	26 (23.4)	**0.013**	62	18 (29.0)	0.129
Total		1022	136 (13.3)			769	126 (16.3)		495	102 (20.6)	

Neg = negative; Sus = serological titer 1:200; Pos = serological titer ≥ 1:800, not vaccinated; Cy = reproduction cycle; N = number of cows; AR = abortion rate; OR = odds ratio; CI = confidence interval; Sig = statistical significance.

**Table 2:** Repeated abortions in three consecutive reproduction cycles in cows from four dairy farms according to their *Neospora* serological status. The distribution of the number of abortions per cow was compared between groups using a Kruskal–Wallis test.

		Number of Abortions Per Cow, N (%)					Total	
Group	N	0	1	2	3	Sig	Abor Ting (%)	Sig
Neg	346	235 (67.9)	100 (28.9)	9 (2.6)	2 (0.6)	ref	111 (32.1)	ref
Sus	87	67 (77)	16 (18.4)	4 (4.6)	0	0.131	20(23.0)	0.117
Pos	62	34 (54.8)	24 (38.7)	3 (4.8)	1 (1.6)	**0.038**	28 (45.2)	0.058
Pos-Vac	60	40 (66.7)	13 (21.7)	7 (11.7)	0	0.558	20 (33.3)	0.881
Total	555	376 (67.7)	153 (27.6)	23 (4.1)	3 (0.5)		179 (32.3)	

N = number of cows; Neg = negative; Sus = serological titer 1:200; Pos = serological titer ≥ 1:800, not vaccinated; Pos-Vac = serological titer ≥ 1:800, vaccinated; Sig = statistical significance; Aborting = the number of cows that aborted at least once.

**Table 3:** Vaccine efficacy in reducing abortion rates of *Neospora*-seropositive cows (titers ≥ 1:800) from four dairy farms in three consecutive reproduction cycles. Cows were vaccinated during the first documented pregnancy with live frozen vaccine, and were not revaccinated in the following reproduction cycles.

Farm	Group	Cyc1 (N)	AR N (%)	Sig	VE (%)	Cyc2 (N)	AR N (%)	Sig	VE (%)	Cyc3 (N)	AR N (%)	Sig	VE (%)	Total Preg (N)	AR N (%)	Sig	VE (%)
Farm 1	NC	27	5 (18.5)			19	5 (26.3)			11	4 (36.4)			57	14 (24.6)		
	Vac	18	3 (16.7)	1	9.72	14	6 (42.9)	0.459	−62.9	4	1 (25.0)	1	31.2	36	10 (27.8)	0.809	−13.1
Farm 2	NC	27	9 (33.3)			15	3 (20.0)			5	0			47	12 (25.5)		
	Vac	18	3 (16.7)	0.308	49.84	14	2 (14.3)	1	28.6	10	0	-	0	42	5 (11.9)	0.115	53.4
Farm 3	NC	55	20 (36.4)			30	6 (20.0)			16	5 (31.3)			101	31 (30.7)		
	Vac	33	3 (9.1)	**0.005**	75	26	0	**0.025**	100	22	4 (18.2)	0.45	41.8	81	7 (8.6)	**<0.001**	71.8
Farm 4	NC	64	19 (29.7)			47	12 (25.5)			30	9 (30.0)			141	40 (28.4)		
	Vac	45	16 (35.6)	0.539	−19.86	34	9 (26.5)	1	−3.7	24	8 (33.3)	1	−11.1	103	33 (32.0)	0.573	−12.9
Total	NC	173	53 (30.6)			111	26 (23.4)			62	18 (29.0)			346	97 (28.0)		
	Vac	114	25 (21.9)	0.136	28.43	88	17 (19.3)	0.603	17.5	60	13 (21.7)	0.408	25.4	262	55 (21.0)	**0.048**	35.1
Total farms 1–3	NC	109	34 (31.2)			64	14 (21.9)			32	9 (28.1)			205	57 (27.8)		
	Vac	69	9 (13.0)	0.007	58.2	54	8 (14.8)	0.354	32.3	36	5 (13.9)	0.229	50.6	159	22 (13.8)	**0.001**	50.2

NC = not vaccinated; Vac = vaccinated; Cyc = reproduction cycle; N = number of pregnancies; R = abortion rate; Sig = statistical significance; VE = vaccine efficacy.

## Data Availability

The data presented in this study are available on request from the corresponding author.
